# Knowledge, attitude, and intentions towards fertility preservation in cancer patients among healthcare workers in Northern India

**DOI:** 10.5935/1518-0557.20210087

**Published:** 2022

**Authors:** Neena Malhotra, Monica Gupta, Anshu Yadav, Perumail Vanamail, Reeta Mahey

**Affiliations:** 1 Department of Obstetrics & Gynecology, All India Institute of Medical Sciences, New Delhi, India

**Keywords:** cancer, fertility preservation, gonadotoxic, oncofertility, healthcare workers

## Abstract

**Objective:**

To study the knowledge, attitude, and intentions towards fertility preservation (FP) in cancer patients among healthcare workers (HCWs) in Northern India.

**Methods:**

The survey discussed in this study was a 23-item structured questionnaire on oncofertility designed based on previous studies on the topic. A link to the questionnaire was sent through WhatsApp to healthcare workers involved in the care of cancer patients. Their responses were analyzed subsequently.

**Results:**

More than a third (37.7%) of the 750 HCWs contacted answered the questionnaire. The group included gynecologists, surgeons, and oncologists. Although 90% of the respondents were aware of the harmful effects of cancer therapy on ovarian function, 76% claimed they had only partial knowledge about fertility preservation (FP). Only a fourth of the respondents were aware of the time needed for oocyte cryopreservation and a third had knowledge of the number of semen samples required for FP. Among HCWs involved in the care of young cancer patients, only 50% reported that they had referred patients for FP. The most common reason for not referring patients for FP was lack of knowledge about FP (43%). More than 90% claimed they wanted to improve their knowledge about oncofertility through continued medical education programs and seminars.

**Conclusions:**

The study emphasizes the need for establishing well-structured networks to improve knowledge about FP among HCWs, so that cancer patients are offered the chance of using their gametes to have children after they have been cured from cancer.

## INTRODUCTION

The incidence of cancer is increasing worldwide. According to data published by the World Health Organization (WHO) in 2018, 9.6 million or one in six deaths were due to cancer (WHO, 2020). GLOBOCAN 2018 reported breast cancer as the most common cancer in the general population (14%) and among females (27.4%), followed by cervical and ovarian cancer (Bray *et al*., 2018). The incidence of breast cancer among younger Indian women is increasing when compared with Western countries (Parmar, 2018). Prevalence of breast cancer is predicted to increase to 35 per 100,000 women in India in 2035 (Mathur *et al*., 2020). With the rising incidence of breast and other cancer types, fertility concerns among young cancer survivors are expected to increase proportionately in the Indian subcontinent.

According to the 2018 Guidelines of American Society of Clinical Oncology (ASCO) and the American Society of Reproductive Medicine (ASRM), healthcare workers (HCWs) involved in the care of adult and pediatric cancer patients should address the possibility of infertility as early as possible before the start of treatment and include the possible options of fertility preservation (FP) in the discussion (Oktay *et al*., 2018). Since time is a crucial element in the management of these patients, fertility counseling and referral to FP should be considered as soon as patients are diagnosed with cancer instead of when they are started on gonadotoxic therapies.

There is an overall lack of awareness among clinicians about the detrimental effects of cancer treatment on fertility, FP options, the timing to refer patients to FP, the times involved in FP procedures, and post procedure implications. Most of the studies performed in Western countries have described poor knowledge, attitude, and awareness of oncofertility among clinicians (Sallem *et al*., 2018; Chehin et al., 2017). Although FP options abound, oncofertility resources have been underutilized and the target population has largely missed out on the chance to have their gametes frozen for purposes of having future parenthood experiences (Woodard *et al*., 2018).

Ours is a tertiary care referral hospital in Northern India that caters to the needs of cancer patients from all over the country. As the number of young cancer survivors in India is on the rise and there is sparse data about oncofertility practices and networks in India, the present cross-sectional study was designed to evaluate the knowledge, attitude, and intentions of HCWs involved in the care of cancer patients regarding the effects of cancer treatment on fertility and fertility preservation options available. The proposed methods to improve the knowledge and awareness of HCWs in this field are also discussed.

## MATERIAL AND METHODS

### Study design

The present cross-sectional study was conducted at a tertiary care center (September 2019 to March 2020) after approval from the Institute Review Board (IEC-566/02.08.2019).

### Questionnaire design

To design the questionnaire, a literature search was done on Pubmed and Google Scholar and articles were searched based on keywords "fertility preservation", "oncofertility", and " knowledge and awareness among clinicians". The next step entailed the organization of a Focus Group Discussion (FGD), during which questionnaire items were developed. Previous studies were reviewed, and questions were designed according to our setup. The questionnaire was tested initially with 10 HCWs (7 gynecologists and 3 oncologists) to check the validity and ease of understanding the questions. Attention was given to keeping the questions in proper sequence and using simple language to allow ease of understanding. After approval, it was sent via mobile messaging application (WhatsApp) to clinicians from the Gynecology, Surgery, and Oncology departments. A brief explanation and the reasons for the survey were delivered in an initial message, which was followed by another message with the link to the questionnaire. Participants answered the questionnaire voluntarily. The questionnaire comprised 23 items and included information about healthcare worker demographics, clinical practice experience, knowledge and awareness of oncofertility as a sub-specialty, and intentions and attitude towards acquiring more knowledge about the field. Participants were asked to provide their email addresses and the rest of the information was anonymized.

### Data collection

The questionnaire was sent to HCWs (gynecologists, surgeons, and oncologists - medical, surgical, and radiation oncologists) involved in the care of cancer patients in Northern India. All the information from the forms completed by the participants was combined in a central database and pre-specified variables were analyzed.

### Statistical analysis

A database was developed using Microsoft Excel and analysis was performed using SPSS IBM version 22.0 (Armonk, NY, IBM Corp.). For normally distributed data, descriptive measures such as mean and standard deviation were calculated. Categorical variables were expressed as frequency and percent values and compared using the Chi-squared/Fisher's exact test. Post-hoc analysis was carried out for multiple comparisons using the Bonferroni correction. For all statistical tests, a two-sided probability of *p*<0.05 was considered for statistical significance.

## RESULTS

A total of 750 HCWs from different specialties involved in the care of cancer patients were sent text messages through WhatsApp with the link to the questionnaire; 287 answered the questionnaire, yielding a response rate of 37.7%.

### Demographic characteristics

Respondent characteristics are shown in Table 1. Ninety-seven of the 283 (34.2%) were gynecologists; 78 (27.5%) were medical oncologists; 58 were surgeons (20.5%); 31 were surgical oncologists (10.9%); and 19 were radiation oncologists (6.7%). About 61.1% of the participants were aged 26-35 years (n=173). The female to male ratio was 1.6:1 (62.8% vs. 37.1%). Almost half of the respondents had been in clinical practice for less than 5 years (47%) and only 70 (24.7%) had been more than 10 years of clinical experience. The most common cancers dealt with by the respondents were gynecological tumors (78.2%), followed by breast, gastrointestinal, urological, hematologic, musculoskeletal, pediatric, and testicular cancer. Almost 83% (236) claimed that they dealt with young cancer patients needing gonadotoxic therapy.

**Table 1.** Characteristics of healthcare workers.

Characteristics	N (%)
Healthcare workers contacted	750
Total responses	283 (37.7%)
Gender	
Male	105 (37.1%)
Female	178 (62.8)
Age (years)	
20-25	16 (5.7%)
26-35	173 (61.1%)
36-45	69 (24.4%)
>45	25 (8.8%)
Specialty	
Gynecology	97 (34.2%)
Medical Oncology	78 (27.5%)
Surgery	58 ( 20.5%)
Surgical Oncology	31 (10.9%)
Radiation Oncology	19 (6.7%)
Experience (years)	
≤5	133 (47%)
6-10	80 (28.3%)
>10	70 (24.7%)

### Knowledge and awareness

More than 90% (n=271) of the respondents were aware that cancer treatment affects future reproduction capabilities. Almost 90% (n=252) were well informed about the association between ovarian function reduction and chemotherapy. Almost 90% (n=253) were aware that among the reproductive organs, the ovaries are more significantly affected by chemotherapy. However, only 44% of the respondents correctly identified cyclophosphamide as the most gonadotoxic chemotherapy agent.

**Figure 1:**
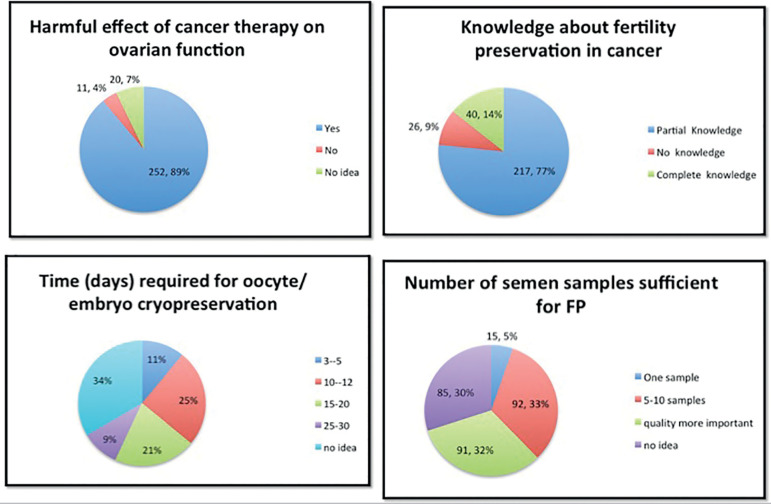
Percentage response regarding knowledge about different options of fertility preservation (FP).

When enquired about their knowledge of FP, almost 77% (n=217) claimed they had partial knowledge about FP in cancer patients (Figure 1). Eighty-six percent (n=245) of the respondents were aware that patients need FP procedures prior to the start of gonadotoxic chemotherapy. But when enquired about the upper age limit for FP in females, only 40% correctly answered that 40 years was the upper age limit.

**Figure 2:**
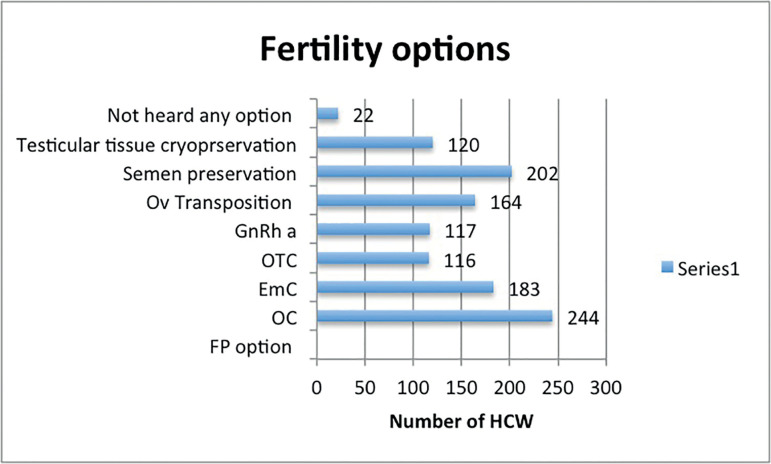
Knowledge regarding various fertility preservation strategies. Abbreviation: Ov transposition, ovarian transposition; OTC, ovarian tissue cryopreservation; EmC, embryo cryopreservation; OC, oocyte cryopreservation; GnRHa, gonadotropin releasing hormone agonist, HCW, health care worker; FP, fertility preservation.

Only 25% (n=71) correctly answered that 10-12 days are needed for oocyte or embryo cryopreservation (Figure 1). Almost two thirds (67%) of the respondents did not know the number of semen samples needed for male fertility preservation (Figure 1). Regarding FP methods, oocyte (86.2%) and semen cryopreservation (71.6%) were among the best known methods (Figure 2).

### Attitude and intentions

Just over half (53%) of the respondents (n=150) routinely referred young cancer patients for FP counseling. The remaining respondents (47%) claimed that lack of knowledge was the main reason why they did not refer patients to FP (43%; n=58). Other common reasons included the inability of patients to afford FP procedures (22.5%, n=30) and the lack of knowledge about referral pathways (16.5%, n=22). Lack of time to counsel patients, marital status (unmarried), and fear of transmitting cancer to their offspring were the least common reasons for not referring patients. More than 90% of the participants (n=260) were desirous of improving their knowledge about FP. The most common paths to improving knowledge about the subject were continued medical education (33.8%), seminars (31.3%), and informative leaflets (23.9%). Almost 97.5% (n=276) of the respondents were willing to join a second follow-up survey. No difference in knowledge was found among clinicians based on years of clinical experience.

## DISCUSSION

The present study found low levels of knowledge and awareness about oncofertility among HCWs in Northern India.

There are global barriers in the field of oncofertility that need to be addressed and overcome by collaboration with professional societies and governments. Another paper published previously by our group found poor overall levels of knowledge among cancer patients about the gonadotoxic effect of cancer therapy and available FP options (Mahey *et al*., 2020).

Although response rates are usually low, most of the studies about knowledge and awareness among clinicians are based on surveys sent by email or social media links (Sallem *et al*., 2018; Chehin *et al*., 2017). A recent global survey with physicians about FP options available to patients had a response rate of only 25% (Rashedi *et al*., 2020). The present study had a higher, although not entirely satisfactory, response rate. The study was stopped at end of six months. As in other studies developed in India (Mahajan *et al*., 2016), the number of female responders was significantly higher than the number of male respondents, possibly because women are more concerned with fertility and parenthood.

Oncologists generally resist discussing FP options, with reasons ranging from the need to treat cancer patients without any delay, a lack of awareness about recent developments in oncofertility, to lack of time (Knight *et al*., 2015). Lack of knowledge was the most commonly reported reason for not referring patients to FP. The findings of this survey may also be extrapolated to the general population of physicians in India, whose knowledge of the subject is lesser than that of physicians working in tertiary care referral hospitals due to limited access to recent updates in this field of medicine. Table 2 shows the gaps in oncofertility referrals and key steps to improve the knowledge of HCWs in our country.

**Table 2.** Gaps in oncofertility services and proposed steps to improve knowledge and awareness in India.

Gaps and concerns in the oncofertility services	Key steps to improve the services
Poor knowledge among oncologists regarding fertility preservation (FP)	1. Establish oncofertility clinics involving oncologists and reproductive medicine (RM) clinicians
	o To plan FP procedures for patients in need.
	o To discuss the type of cancer, survival rate, risk of gonadotoxicity from cancer therapy, desire for fertility preservation, time available, physical and medical fitness of patient to undergo FP procedure.
	2. Organize FP seminars and continued medical education (CME) and produce leaflets so that information can be disseminated to cancer clinicians at a national level.
Lack of time	Involvement of RM clinicians/fellows to counsel patients at the time of initial diagnosis so that reproductive concerns may be taken care of.
	o Other staff such as social workers and nurses can be trained to discuss fertility issues with patients. They may counsel patients in preliminary interviews and refer patients in need.
Financial concerns	The procedure of gamete/embryo cryopreservation may be available at minimum or free of cost to patients, especially for those unable to bear the cost of the procedures.
Patient concerns	Detailed patient counseling by the oncologist and RM clinician at the time of cancer diagnosis to allow timely referral, so that patient and family concerns are addressed and FP is performed without delaying cancer treatment.
Building awareness at a national level	Use social media platforms to provide an easy and approachable network to manage these patients. Support of professional societies and national bodies can be sought to organize conferences at a national level for wider dissemination of information.
Bridging the gap between cancer and fertility	Establish an Oncofertility Consortium
	o Multidisciplinary team comprising medical specialists (e.g., oncologists, hematologists, reproductive endocrinologists, urologists, surgeons, pathologists) and healthcare staff including nurses and genetic, mental health and social counselors, and the embryology/andrology lab team
	o Establishing and maintaining communication between the various stakeholders

Since lack of time has been reported as of the major reasons for not referring patients to FP, other HCWs such as nurses and counselors who also come in contact with young cancer patients during treatment may play an important role at advising patients about the effects of cancer therapy, educating them about FP, scheduling consultations with fertility clinicians, and discussing financial implications.

Our survey found that HCWs had little knowledge about the time needed for oocyte/embryo freezing and the number of semen samples required for FP. There is a need to develop structured networks to improve knowledge about oncofertility in each level, so that timely referral is made possible. We need to explore the social, ethical, religious, and financial factors affecting FP decisions among patients. Financials factors are typically important in low-to-middle-income countries (LMIC) like ours, since FP procedures are not offered free of charge or covered by insurance, which means that families must bear the expense (Rashedi *et al*., 2020). Even in cases of timely referral to FP, some patients cannot undergo the procedure due to financial constraints and the short window they are allowed to make the required monetary arrangements.

The limitations of the study include the small size of the sample and the low response rate of participants contacted. We were unable to get responses from physicians of other specialties such as hematology. The study found overall meager knowledge about FP among HCWs and emphasized the need to spread awareness through different social media platforms and provide for an easy and approachable network to manage these patients. Support of professional societies and national platforms are desirable to establish oncofertility care so that the dream of parenthood may come true for cancer survivors.
